# The prevalence of human papillomavirus among women in northern Guangdong Province of China

**DOI:** 10.1038/s41598-022-17632-y

**Published:** 2022-08-03

**Authors:** Wenbo Huang, Hongyan Xu, Hongbo Hu, Dingmei Zhang, Yulan Liu, Yanle Guo, Fengjin Xiao, Weijuan Chen, Zhanzhong Ma

**Affiliations:** 1 Yuebei People’s Hospital, Shantou University Medical College, Shaoguan, 512026 China; 2 School of Public Health, Sun Yat-Sen University, Guangzhou, 510080 China

**Keywords:** Diseases, Molecular medicine

## Abstract

Globally, cervical cancer, whose etiologic factor is Human papillomavirus (HPV), is the third most common cancer among women. In cervical cancer screening, HPV testing is important. However, the prevalence of HPV in northern Guangdong Province has not been conclusively determined. A total of 100,994 women attending Yuebei People's Hospital Affiliated to Shantou University Medical College between 2012 and 2020 were recruited. HPV was tested by a polymerase chain reaction (PCR)-based hybridization gene chip assay. The prevalence of HPV among these women was established to be19.04%. Peak prevalence was observed in women aged 40–49 (7.29%). Besides, the prevalence of single-type HPV infection (14.46%) was significantly high, compared to multiple-type infection (4.58%) (*p* < 0.01), while the prevalence of high-risk HPV infection (19.97%) was significantly higher than that of low-risk genotypes (5.48%) (*p* < 0.01). The most prevalent high-risk genotypes were HPV52 (4.16%), HPV16 (2.98%), HPV58 (2.15%), HPV53 (1.58%) and HPV68 (1.34%). HPV co-infection with up to 10 genotypes was reported for the first time. Our findings suggested a high burden of HPV infections among women in northern Guangdong. Establishing the prevalence and genotype distribution characteristics of HPV infections in the region can contribute to cervical cancer prevention through HPV vaccination.

## Introduction

Globally, cervical cancer which can be caused by the major oncogenic types of HPV, is the third most common cancer in women^1,2^. Therefore, HPV vaccination can significantly reduce the risk of cervical cancer^3^. HPV is a circular double-stranded DNA virus that mainly infects epithelial cells in the skin and mucosal cells. The International Agency for Research on Cancer (IARC) reports that there are more than 200 HPV genotypes, among which, about 40 genotypes infect the genitourinary system while 20 genotypes are associated with cancer development^4^. Most HPV infections have no obvious clinical symptoms, and the spontaneously resolve^5^. Based on their risk of causing cancer, HPVs are classified as high-risk HPV (HR-HPV) or low-risk HPV (LR-HPV). HR-HPV can cause cervical cancer, with HPV 16/18 exhibiting the highest risk of cancer development. It usually takes 10–20 years for HPV infection-driven lesions to progress to malignancy^6–8^.

There are regional differences in HPV prevalence and genotype distribution, which explains the different types of vaccines^9,10^. The currently licensed HPV vaccines include bivalent vaccines (HPV16/18), quadrivalent vaccines (HPV16/18/6/11), andnine-valent vaccines (HPV16/18/6/11/31/33/45/52/58)^11,12^. Therefore, to select a suitable vaccine, it is important to understand local HPV incidences and subtype distribution.

This study aimed to investigate the prevalence and genotype distribution of HPV in northern Guangdong province, so as to provide a scientific basis for early screening strategies and vaccination for cervical cancer in the region.

## Materials and methods

### Subjects

A total of 100,994 women attending the Physical Examination Center and Department of Gynecology of Yuebei People’s Hospital between January 1st, 2012 and December 31st, 2020 were enrolled in this study. The inclusion criteria were: (1) Aged 18–75 years; (2) All participants resided in northern Guangdong Province; (3) No history of sexual life or vaginal medication in the past one week; (4) Willingness to undergo an HPV test and participate in the present study, All participants signed informed consent. This research was approved by the Ethics Committees of Yuebei People’s Hospital (KY-2019-083, Approved October 10, 2019). All methods were performed in accordance with relevant guidelines and standard operating procedures (SOPs).

### Specimen collection

Gynecological practitioners collected the cervical specimens using cervical brush. First, the cervix was exposed using a vaginal dilator, secretions were wiped using a cotton swab, the cervical brush was extended into the cervix and rotated 4 to 5 times in a clockwise direction to obtain a sufficient amount of cervical epithelial cells. Then, the brush was placed in a vial containing the cell preservation solution (Yaneng Biotechnology, Ltd., Shenzhen, China). Cervical samples should be stored at room temperature for no more than 12 h and at 4 °C for no more than 7 days after collection. Specimens were sent to the department of clinical PCR laboratory in the hospital for testing.

### DNA extraction

DNA from exfoliated cervical cells was extracted using a DNA extraction kit (Yaneng Biotechnology, Ltd., Shenzhen, China). Samples on the cervical brushes were eluted; the elution was transferred to a 1.5 ml centrifuge tube, centrifuged at 13,000 rpm for 10 min, and the supernatant discarded. To the cell pellet, 50 µl of the lysis buffer was added to suspend the pellet, incubated at 100 °C for 10 min, and centrifuged at 13,000 rpm for 10 min, to obtain DNA.

### HPV DNA testing

HPV DNA PCR amplification and genotyping were performed using the HPV genotyping Kit (Yaneng Biotechnology, Ltd., Shenzhen, China) according to the manufacturer’s instructions. The test kit was approved by the National Medical Products Administration (NMPA, No.20193401918). The test included 23 type-specific oligonucleotides, designed to detect 17 HR-HPV genotypes (HPV 16, 18, 31, 33, 35, 39, 45, 51, 52, 53, 56, 58, 59, 66, 68, 73 and 82) together with 6 LR-HPV genotypes (HPV6, 11, 42, 43, 81 and 83). Amplification was performed using the BIOER Genetouch PCR amplification instrument. The amplification system consisted of 5 μl HPV-DNA and 20 μl reaction system. Reaction conditions were: 50 °C for 15 min, denaturation at 95 °C for 10 min, denaturation at 94 °C for 30 s, annealing at 42 °C for 90 s, elongation at 72 °C for 30 s, 40 cycles, elongation at 72 °C for 5 min, and storage at 4 °C. HPV genotyping was performed by hybridization in a YN-H16 thermostatic hybridization instrument. The final result was determined by directly observing color reactions on the chip. The negative control showed no color reaction at all sites except the blue dot on the internal control (IC) site. The positive control must be color reaction of the positive HPV genotype and IC site (shown in blue), and the genotype is read according to the site of the blue spot on the chip.

### Statistical analysis

Data analysis was performed using SPSS22.0 software (IBM, USA). Data were presented as mean ± standard deviation or frequency and percentage for numerical or discrete variables, respectively. The Chi-square test was used to determine significant differences between groups, and differences were considered statistically significant at *p* < 0.05.

### Ethics statement

This research was approved by the Ethics Committees of Yuebei People’s Hospital.

## Results

### Age-specific HPV prevalence

**Figure 1:**
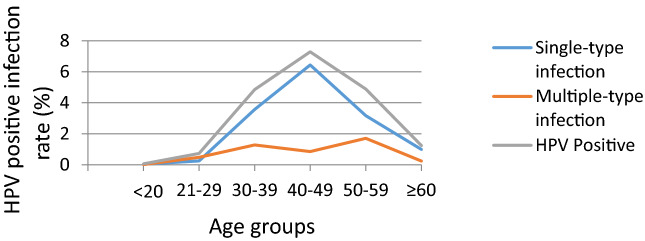
HPV infection rate in different age groups.

A total of 100,994 women visited the Yuebei People's Hospital and subjected to HPV DNA testing. The basic characteristics of study participants are shown in Table 1. It was established that 19,233 (19.04%) of the women had HPV infections, with most of the infections occurring in women of the 40–49 age group (7.29%). Single-type HPV infections were common among women aged 40–49 years (6.44%), while multiple-type HPV infections were common among women aged 50–59 years (1.71%). Single-type HPV infections (14.46%) were more common than multiple-type infections (4.58%), (*p* < 0.01; Table 2 and Fig. 1).

**Table 1:** Basic characteristics of study participants (N = 100,994).

Characteristic	Case (n)	Percentage (%)
Age in years, mean (SD)	42.60 (10.53)	
**Age (years)**		
≤ 20	225	0.22
21–29	11,723	11.61
30–39	26,150	25.89
40–49	38,098	37.72
50–59	20,064	19.87
≥ 60	4734	4.69
**Education level**		
Primary	10,821	10.71
Secondary	36,069	35.71
Higher	54,104	53.57
**Marital status**		
Never married	7921	7.84
Married	89,112	88.24
Divorced/widowed	3961	3.92
**Number of children**		
0	7622	7.55
1	57,166	56.60
2	32,394	32.08
≥ 3	3811	3.77
**Alcohol consumption**		
Ever		
Never		
**Tobacco**		
Never	95,485	94.55
< 7 cigarettes a week	3673	3.64
≥ 7 cigarettes a week	1836	1.82
Age of first sexual intercourse; mean (SD)	22.8 (3.1)

**Table 2:** Single and multiple type infections of HPV in different age groups.

Age (years)	N	Single-type infection		Multiple-type infection		HPV Positive		95%CI	*P* Value
		No	(%)	No	(%)	No	(%)		
≤ 20	225	44	0.04	13	0.01	57	0.05	0.04–0.07	< 0.001
21–29	11,723	248	0.25	490	0.48	738	0.73	0.70–0.76	< 0.001
30–39	26,150	3607	3.57	1296	1.28	4903	4.85	4.54–5.16	< 0.001
40–49	38,098	6504	6.44	857	0.85	7361	7.29	7.01–7.56	< 0.001
50–59	20,064	3201	3.17	1725	1.71	4926	4.88	4.57–5.19	< 0.001
≥ 60	4734	1002	0.99	247	0.24	1249	1.24	1.03–1.44	< 0.001
Total	100,994	14,605	14.46	4628	4.58	19,233	19.04	18.80–19.30	< 0.001

### HPV genotype distribution

**Figure 2:**
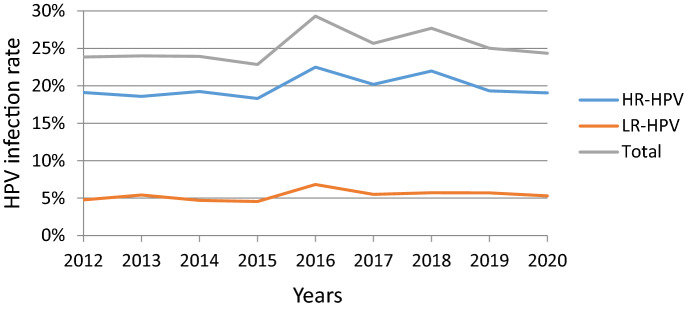
HPV infection rate from 2012 to 2020.

**Figure 3:**
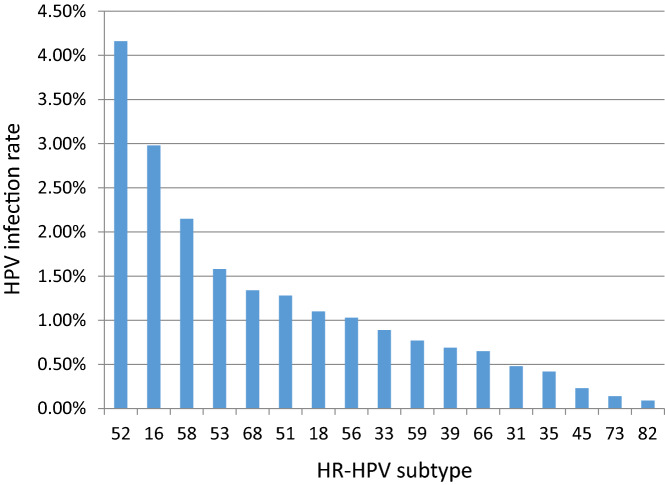
Distribution of HR-HPV subtypes infection.

**Figure 4:**
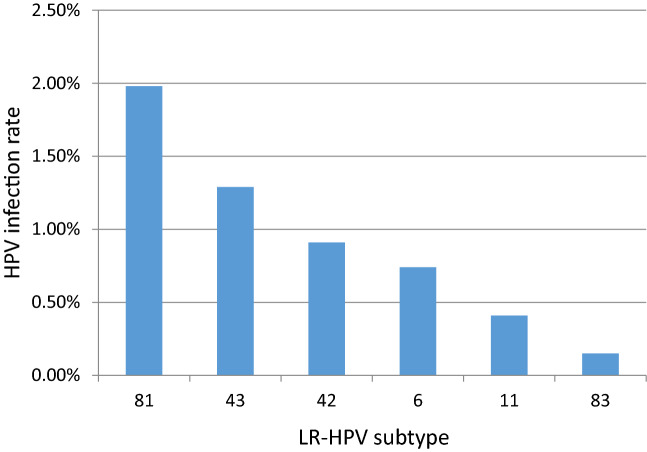
Distribution of LR-HPV subtypes infection.

A total of 25,710 positive HPV genotypes were detected in samples from the 100,994 women (Since some women were infected with multiple genotypes, so the total number of positive HPV genotypes was greater than the number of infected women). Twenty-three HPV genotypes were identified (Table 3 and Fig. 2). The proportions of HR-HPV and LR-HPV infections were 19.97% and 5.48%, respectively. The top ten most prevalent HR-HPV genotypes were HPV52, 16, 58, 53, 68, 51, 18, 56, 33, and 59, with corresponding proportions of 4.16%, 2.98%, 2.15%, 1.58%, 1.34%, 1.28%, 1.10%, 1.03%, 0.89% and 0.77%, respectively (Fig. 3). The LR-HPV genotypes included HPV81, 43, 42, 6, 11 and 83, with corresponding proportions of 1.98%, 1.29%, 0.91%, 0.74%, 0.41% and 0.15%, respectively (Fig. 4). The distribution of HR-HPV in high-grade lesions cervical and invasive cervical cancer is shown in Table 4.

**Table 3:** Positivity of HPV genotypes from 2012 to 2020.

HPV genotype	2012 (N = 5280)	2013 (N = 6566)	2014 (N = 8497)	2015 (N = 9204)	2016 (N = 11,720)	2017 (N = 13,330)	2018 (N = 14,356)	2019 (N = 17,112)	2020 (N = 14,929)	Total (N = 100,994)	Positive rates %	*P* Value
**HR-HPV**	1008	1220	1634	1684	2635	2689	3153	3306	2844	20,173	19.97	< 0.001
16	311	283	257	263	379	359	406	404	345	3007	2.98	< 0.001
18	92	75	105	104	136	119	169	191	124	1115	1.10	< 0.001
31	38	51	38	41	67	52	83	68	43	481	0.48	< 0.001
33	63	89	91	58	119	116	110	141	107	894	0.89	< 0.001
35	24	39	39	34	53	56	73	51	59	428	0.42	< 0.001
39	3	12	70	59	74	107	123	131	119	698	0.69	0.061
45	6	10	23	19	25	34	37	47	36	237	0.23	< 0.001
51	9	35	95	114	156	184	224	274	199	1290	1.28	0.436
52	120	171	273	355	629	639	676	686	657	4206	4.16	< 0.001
53	14	38	132	138	230	249	272	266	258	1597	1.58	< 0.001
56	57	77	96	92	123	113	144	165	169	1036	1.03	< 0.001
58	154	200	169	148	272	283	317	326	298	2167	2.15	0.333
59	24	30	58	66	102	101	125	153	119	778	0.77	< 0.001
66	38	34	52	46	71	75	117	124	98	655	0.65	0.003
68	51	65	131	132	175	175	228	228	171	1356	1.34	0.058
73	4	8	5	10	16	19	28	33	16	139	0.14	< 0.001
82	0	3	0	5	8	8	21	18	26	89	0.09	0.075
**LR-HPV**	251	356	399	418	798	732	821	973	789	5537	5.48	0.689
6	40	50	59	61	108	105	117	114	94	748	0.74	< 0.001
11	29	42	41	51	49	46	48	59	47	412	0.41	0.172
42	31	58	64	67	111	125	140	167	158	921	0.91	0.001
43	144	193	225	103	134	97	126	160	122	1304	1.29	0.028
81	0	0	0	121	375	337	365	448	353	1999	1.98	< 0.001
83	7	13	10	15	21	22	25	25	15	153	0.15	< 0.001
Total	1259	1576	2033	2102	3433	3421	3974	4279	3633	25,710	25.46	< 0.001

**Table 4:** The HR-HPV genotype distribution in HSIL and ICC patients.

HR-HPV genotype	HSIL		ICC	
	Case (n)	Percentage (%)	Case (n)	Percentage (%)
16	312	27.2	74	68.5
58	250	21.8	6	5.6
52	188	16.4	11	10.2
33	117	10.2	0	0
31	49	4.3	1	0.9
18	43	3.8	12	11.1
51	32	2.8	0	0
56	26	2.3	1	0.9
39	24	2.1	0	0
66	21	1.8	0	0
53	20	1.7	0	0
68	20	1.7	1	0.9
59	15	1.3	2	1.9
82	13	1.1	0	0
35	8	0.7	0	0
45	6	0.5	0	0
73	1	0.1	0	0
Total	1145	100	108	100

HSIL: High-grade squamous intraepithelial lesion.

ICC: Invasive cervical cancer.

### Distribution of single and multiple HPV infections

**Figure 5:**
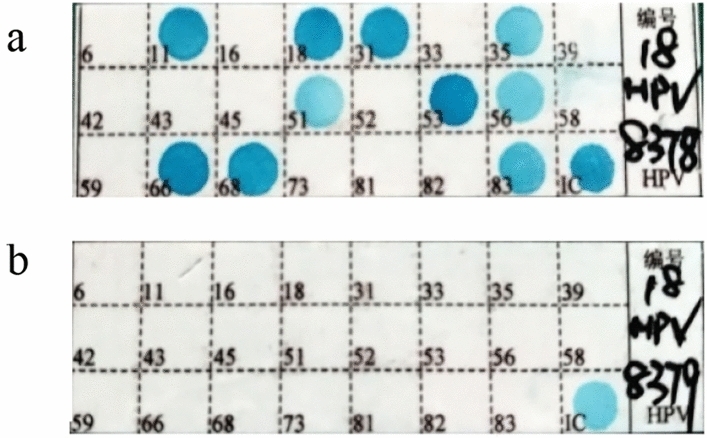
(**a**) HPV positive coinfection with 10 subtypes; (**b**) HPV negative ybridization.

Single, double, and multiple-type HPV infections accounted for 75.94%, 17.52% and 4.47%, respectively (Table 5). Single-type infection was more common than multiple-type infection (*p* < 0.01). HPV co-infection with up to 10 subtypes was discovered for the first time (Fig. 5).

**Table 5:** Distribution of single and multiple-type HPV infections.

Number of infection types	Positive, No	Composition ratio (%)
1 Type	14,605	75.94
2 Types	3370	17.52
3 Types	860	4.47
4 Types	291	1.51
5 Types	58	0.30
6 Types	27	0.14
7 Types	13	0.07
8 Types	5	0.03
9 Types	2	0.01
10 Types	2	0.01
Total	19,233	100.00

## Discussion

In this study, we established that the HPV infection rate among women in northern Guangdong was 19.04%, with the infection rate being highest among women aged 40–49 (7.29%). Single-type and HR-HPV infections were the most prevalent form. The most prevalent HR-HPV infections were HPV52, 16, 58, 53, 68, 51, 18, and 56 while LR-HPV infections were mainly HPV81 and 43. We also report, for the first time, HPV co-infection with up to 10 genotypes in two cases.

Studies have reported differences in HPV prevalence and genotype distribution in different countries^13^. According to a comprehensive meta-analysis involving over 1 million women, the global HPV prevalence is estimated to be 11.7%. Sub-Saharan Africa (24.0%), Eastern Europe (21.4%), and Latin America (16.1%) have the highest prevalence^14^. In Japan, the prevalence of HPV is 25%, with HPV52, 16, and 58 being the most common genotypes^15^. In Korea, the prevalence of HPV is 16.71%, with HPV 53, 58, and 52 being the most common genotypes^16^. A previous meta-analysis reported that HPV infection rate among women in mainland China is 19.0%, with HPV16, 52, and58 being the most common genotypes^17^. These types are also the most common types of CIN in China^18^. This contrasts with the situation in Western countries, where HPV16 and 18 infections are prevalent^19,20^.

In China, there are regional differences in HPV prevalence and genotype distribution. Population-based screening revealed that the HPV infection rate in China ranges from 9.9% to 31.94%^21–24^. Among the sampled regions, Haikou (31.94%), Beijing (21.06%), and Guangdong (20.02%) have the highest HPV incidences. Our results are consistent with HPV distribution characteristics previously reported in China^25–27^. The overall HPV infection rate is high and single-type as well as high-risk infections are more common. However, the top five genotypes are slightly different. For instance, the main genotypes in Meizhou^28^, were HPV 16, 52, 58, 18, and 81, while in northern Guangdong, they were HPV 52, 16, 58, 53 and 68. In short, the top three in most regions of China were HPV 52, 16, and 58.

HPV screening and vaccination are the most effective measures for preventing cervical cancer. China's cervical cancer screening program adopts a combined method for HPV and cytology (TCT) screening, which can maximize screening Sensitivity and specificity to improve the detection rate. The program is led by the government, with the participation of social groups, medical institutions, third-party testing institutions, etc. It is completely free for the subjects, voluntary, and covers women aged 35–64. In short, cervical cancer screening has a long way to go, and various efforts are being made to explore solutions with Chinese characteristics. Most developed countries have adopted vaccination as their main preventive strategy. Therefore, if these prevention strategies can be promoted in developing countries, thousands of lives can be saved. Currently, the three available HPV vaccines are all prepared according to the existing epidemiological characteristics of HPV in Western countries. At the same time, there is controversy with regards the vaccination age. The American Cancer Society (ACS) recommends that HPV vaccination be performed at 9 to 12 years old to reduce cervical cancer incidences. However, ACS does not endorse the 2019 Advisory Committee on Immunization Practices recommendation for shared clinical decision for adults aged 27 to 45 years potential of vaccination^29^.

China approved the importation of bivalent vaccines in 2016. Nationally, the HPV vaccination rate for women is only 11.0%^30^, and the vaccination situation is not optimistic. HPV vaccination is expensive, and uncertainties regarding the side effects of the vaccine are the main reason for reluctance. In this study, we found that the HPV infection rate remained stable without any downward trend from 2012 to 2020. Therefore, China needs to develop HPV vaccines that are suitable for the Chinese population, while putting into consideration the characteristics of vaccine genotypes and the age of vaccination, further localization, reducing costs, and including government free vaccination programs to reduce the incidences of HPV infections and cervical cancer.

## Conclusions

For the first time, we report on prevalence and subtype distribution of women HPV in northern Guangdong, which is of great significance for guiding the formulation of local vaccination and other prevention strategies. However, epidemiological investigation of HPV requires multi-center and long-term monitoring, especially evaluation of the effects after vaccination. Besides, persistent HR-HPV and multiple-type infections as well as cancer outcomes should be investigated further.

## Data Availability

The original data can be obtained by contacting the corresponding author.
